# Selection of presale models for innovative products considering channel preferences and valuation differences

**DOI:** 10.1371/journal.pone.0299945

**Published:** 2024-03-28

**Authors:** Manman Jiang, Liping Qin, Wenjin Zuo, Qiang Hu

**Affiliations:** Shanghai University of Finance and Economics Zhejiang College, Jinhua, China; Wuhan Textile University, CHINA

## Abstract

To reduce financial pressure and operational risk, and improve match between supply and demand, an increasing number of enterprises are adopting presales to launch new products. In this context, this paper investigates three presale models for innovative products, namely, the no-presale model, the manufacturer presale model and the retailer presale model. A Hotelling model is used to describe the impact of channel preferences and valuation differences on the two-stage competition between innovative products. Aiming at evaluating the purchase behavior of consumers under three presale modes, a game optimization model is established to analyze the presale decision problem for innovative products under different presale entities. The research shows that: (1) Compared with no-presale, presales can help enterprises obtain more profits. The overall profit of the supply chain is optimal under the retailer presale mode. (2) When the difference in channel preferences is small, the manufacturer obtains the greatest profit by choosing the direct presale model. In contrast, the selection of different presale entities has a significant impact on product sales and supply chain enterprise profits. At this point, the manufacturer should choose the retailer presale model. (3) When the product valuation discount is high, the manufacturer can increase the spot and wholesale prices to induce consumers to choose the presale method to purchase the product. In the opposite situation, the manufacturer should lower the presale price to improve the presale utility of consumers and encourage them to participate in the presale. These conclusions provide more targeted suggestions for enterprises to formulate presale strategies, which can help them grasp market demand and improve market competitiveness.

## 1. Introduction

The popularization of information technology has not only changed people’s way of life but also had a profound impact on various industries. Especially in the field of product research and development, the application of information technology has led to an unprecedented increase in the speed of product iteration. This means that enterprises can launch new products faster to meet the changing needs of the market. For example, the NIO EC6 was launched in May 2023, and only a month later, the EC7 was launched. In a competitive market environment, enterprises that can quickly respond to market changes and launch new products are often able to take the lead and gain more market shares. However, due to the diversity and complexity of the market demand information for innovative products, manufacturers face greater uncertainty when forecasting market demand, which ultimately leads to an imbalance between market supply and demand [1].

To cope with market uncertainty and improve the match between product supply and demand, enterprises are adopting presale methods to launch new products. For example, Apple officially opened the presale of the iPhone 14 series products on September 8, 2022. According to e-commerce market testing data, compared with that of the iPhone 13 series, the presale volume of the iPhone 14 series increased by 7% in the same period, and sales increased by 17% year-on-year. In particular, sales of the Pro series increased by 56% compared to those in the same period. Tesla launched the Cybertruck at the end of 2019, which was favored by many consumers before mass production began. The presale orders continued to increase and exceeded 1.94 million as of July 22, 2023. Presale not only helps enterprises produce on demand and reduce the financial pressure and operational risk caused by inventory backlogs but also accurately predicts spot demand. For consumers, presale provides them with the opportunity to acquire and lock in products in advance to avoid regret due to higher prices or out-of-stock issues during the spot sale period [2]. Therefore, as an emerging sales model, the presale model has been widely applied in many industries. It not only opens new markets for new products but also provides consumers with more purchasing options. Faced with the choice of multiple shopping methods in the market, consumers will conduct rational analysis and consider the influence of various factors, such as the price of the product, the out-of-stock rate, and the timeliness of innovation on their purchase decisions. They will choose the appropriate purchasing method and timing according to their needs and the principle of utility maximization [3]. However, due to the uniqueness and novelty of innovative products, consumers are uncertain about product valuation, which increases the difficulty of evaluating product value and performance. Moreover, consumers’ valuation of products has a strong subjective component. Due to the influence of factors such as product availability and innovation timeliness, there are certain differences in the valuation of innovative products among different consumers, which in turn affects their purchasing decisions regarding spot products [4].

Because of the normalization of online shopping, direct presale has become the preferred choice for enterprises. For example, the Huawei Mate60Pro was launched for presale on Huawei Mall’s official website on September 8, 2023. Direct presales not only make Huawei’s products sell out in a short time but also provide consumers with more shopping convenience. Although the direct sale model has shown advantages, some manufacturers choose to cooperate with traditional retailers and resell their products to retailers who carry out presales. For example, Guangzhou Pharmaceuticals Baiyunshan carries out presale "second to kill" activities in offline stores, such as Jianmin Pharmaceuticals and Caizhilin, to guide traffic to the stores, which then provide offline experience and shopping services. Due to the differences in the amount of information held by different enterprises, the status and discourse power of enterprises in decision-making are obviously different [5]. Retailers hope that the presales can provide more benefits, while manufacturers weigh the relationship between presale costs and benefits to make decisions. The difference in the presale model has a significant impact on the operational efficiency of the supply chain and product sales. Therefore, it is highly important to study manufacturer’s presale mode selection for scientific and effective channel management.

The booming development of e-commerce has created conditions for manufacturers to conduct both online and offline presale activities. The presale model that combines the internet and traditional markets has become an important means for enterprises to resist risks and achieve supply and demand match [6]. However, due to the lack of face-to-face communication and interaction in online channels, consumers are unable to directly access physical products. In contrast, offline channels provide more opportunities for real-time interactive shopping experiences with salespeople and other consumers to build trust relationships with physical stores. Therefore, consumers prefer offline channels to be able to better understand and experience product. Therefore, the impact of channel preference on enterprise presale decisions and consumer purchasing behavior cannot be ignored.

In view of this, this paper raises the following questions:

RQ1. How does the selection of different presale entities affect the decision-making processes and profits of supply chain enterprises for the presale of innovative products?
RQ2. Under different channel preferences, which presale model will make the overall decision optimal?
RQ3. What is the impact of product valuation differences on the choice of presale models, and how does this choice affect the presale decisions and profits of enterprises?

To solve the above problems, this paper adopts a linear Hotelling model to study the presale decision-making for innovative products under different presale entities. The paper constructs presale decision models for innovative products with no-presale, manufacturer presale and retailer presale. By comparing the equilibrium results of different presale models and analyzing the influence of channel preferences and valuation differences on supply chain presale decisions, we explore the relationships between channel preferences and presale entity selection, as well as the manufacturer’s choice of presale model under different channel preferences. The purpose of this paper is to analyze the impact of enterprise presales on the supply chain and how the choice of different presale entities can achieve a win-win situation for supply chain enterprises and to provide decision support for the development of manufacturers’ presales activities.

The remainder of this paper is organized as follows. The Literature review section reviews the relevant literature and illuminates the gaps in this research area. The Problem description and basic assumptions section describes the framework of the model, the basic assumptions, and the associated notations. The Modeling and analysis section constructs three game models, solves the models, and analyzes the equilibrium results. The Numerical analysis section describes the data simulations and sensitivity analysis. The Conclusion section summarizes the conclusions and provides some managerial insights. All the proofs are shown in the S1 Appendix.

## 2. Literature review

The related literature includes three main streams of research: presale strategy, product valuation differences, and dual-channel supply chains.

### 2.1 Presale strategy

With the development of big data technology, manufacturers are becoming increasingly aware of the importance of the presale strategy. Through presale, firms can obtain consumer feedback and preference information before a product is launched to better meet market demand and optimize product design. This strategy can not only help manufacturers reduce production costs and inventory risks but also improve the market competitiveness of products [7–9]. In the early stage, scholars focused mainly on the presale strategy of service goods. Bigne et al. [9] analyzed the relationship between different channel styles and multiple destinations on the dynamics of hotel prices under the advance booking model. Noori-daryan et al. [10] examined the behavior of two complementary enterprises in the airline and hospitality industries using advance reservation policies when partial refunds were allowed. Current research on presale strategy focuses primarily on presale pricing [11–13], capacity and inventory [14–16], return policy and consumer presale regret [17, 18] and other presale-related issues [19–21]. Zhang et al. [11] shows that under the presale mode of new products, the influence of reference price on the presale decision and pricing. Duary et al. [12] study the use of immediate price discounts by manufacturers against prepayment with the purpose of incentivizing retailers to preorder goods and reducing market uncertainty. Lu and Wang [13] analyze the pricing problem of new products for consumers with social learning ability under the presale mode. Yu and Yan [14] examine the manufacturer’s choice of a presale strategy to sell seasonal products to consumers under uncertain supply and demand. Quan and Cho [15] incorporate the demand information into inventory allocation during the presale period to investigate retailers’ inventory allocation and pricing strategy. Yu et al. [16] investigate consumer valuation bias of innovative products in presale and spot sale periods and how consumers respond to the valuation bias of products in the presale period and the spot sale period when facing different return costs. Jiang et al. [17] explore the impact of consumer out-of-stock regret on product presale pricing and retailer profitability. Wang et al. [18] measure consumer utility based on time preference and compare four presale strategy models to maximize seller revenue. Zou [19] reports that in the face of market competition and information asymmetry, retailers adopt a reverse presale model that promotes the retailer’s market share while increasing the enterprise’s profits and order quantity. Yang et al. [20] consider retailer fairness concerns and construct three game models to analyze the impact of the presale scale and consumer green preferences on the optimal supply chain decision. Although scholars have conducted in-depth research on presale strategies from multiple perspectives, in the current literature they have focused mainly on the presale model of a single entity. However, in the actual presale process, the manufacturer can either choose direct channel to carry out presales or hand over the presale right to a retailer. The choice of presale entities will have a great impact on the product pricing, sales volume, and profits of supply chain enterprises.

### 2.2 Product valuation difference

In recent years, more scholars have begun to pay attention to the issue of differences in product valuation in presales. Yan et al. [21] emphasize the effect of the correlation between consumer valuation and production capacity on enterprise decision-making. When the correlation between them is weak, enterprises motivate consumers through presale discounts; in the opposite case, enterprises control the presale volume for premium presale. Zeng et al. [22] study the internal relationship between enterprise risk aversion and product technology sharing behavior when consumers are uncertain about product evaluation. Fang et al. [23] consider the characteristics of consumer market segmentation and valuation heterogeneity and examined the problem of presale strategies and decision-making for retailers selling products. Yi et al. [24] study monopoly-type digital goods firms using product displays to address consumer valuation uncertainty, which not only optimizes product antipiracy measures but also increases product profitability. Guo et al. [25] analyze the high return rate of products caused by the valuation deviation of online products, and online retailers use the wordless shopping service to solve the product valuation problem. Zhang et al. [26] investigate the impact of consumer expected regret on the presale hybrid bundling strategy when consumer valuations are uncertain.

Most scholars focus their research on the differences in the valuation of ordinary products, while relatively few studies evaluate innovative products. In fact, consumers are usually willing to pay higher prices for innovative products because they believe these products will provide a better experience or solve their problems. Moreover, consumers have high requirements for the timeliness of innovative products, and they hope to obtain these products as soon as possible. This pursuit of pursuing innovativeness and timeliness makes the presale valuation of innovative products different from that of ordinary products [27]. Zhang et al. [28] study how strategic consumers respond to their presales strategies in the face of competitive retailers selling homogeneous new products. Wu et al. [29] find by weighing presale income and loss that by exploiting the uncertainty in the presale valuation of new products, presales at a discount could both increase presale sales and reduce inventory costs. Du et al. [30] consider the impact of manufacturers disclosing or concealing the future market prices of new products during the presale stage on the optimal presale and ordering strategies for retailers selling both innovative and marketed products. Zhang et al. [31] demonstrate that demand information about new products and consumer valuation uncertainty have a significant impact on consumer purchasing behavior and retail operations. Therefore, for the valuation of innovative products, we cannot simply apply traditional valuation methods; rather, we need to deeply consider their particularity and consumer needs in detail to more accurately understand and reveal the economic value of innovative products.

### 2.3 Dual channel supply chain

The rapid development of e-commerce has promoted the diversification of consumer demand, and the dual-channel sales model has gradually become a trend. Enterprises use both online and offline channels to sell. This dual-channel sales model provides enterprises with more sales opportunities and flexibility but also creates more challenges and complexity [32, 33]. In the face of multiple shopping channels, consumers are more likely to accept offline channels, which allow them to not only experience products but also obtain more product information. The online channel is less acceptable because consumers cannot directly touch physical products [34]. Li et al. [35] consider three different coupon distribution models for dual-channel promotions and compare the role of coupons in product pricing and channel competition. Zhang et al. [36] emphasize the dynamic pricing of two-stage dual-channel supply chain for manufacturers and retailers and employ cost sharing contracts to improve supply chain performance. Xu et al. [37] study the influence of cost models of consumers with different channel preferences on the optimal channel structure in sales activities. Although little research has been conducted on dual-channel presale strategies, some scholars have begun to pay attention to this topic. Guo et al. [38] explain the impact of changes of free riding on channel pricing, presale service strategies and profits under three different scenarios: Stackelberg competition, Bertrand competition, and channel integration. Zhao and Li [39] discuss market segmentation and price discounts and suggest manufacturers purposefully guide consumers to choose channels to maximize their profits. Wu et al. [40] conducted a study on the impact of the consumption of fresh products in circulation on enterprise presale. Their study demonstrates that under the presale efforts of supply chain members, direct presales can increase enterprise profits. Wang et al. [41] use dynamic programming to construct three different two-stage trade-in pricing models and find that the optimal price is determined by the proportion of old customers, the discount coefficient, and product innovation level. Although the above studies address dual-channel presales, they focus only on the influence of channel selection on product pricing strategies and lack in-depth discussion on the impact of presale entities by channel selection. In practice, when enterprises choose different channels for presale, it will not only affect the price of the product but also change the choice of the presale entities. Therefore, this paper aims to fill this research gap and provide a useful reference and guidance for enterprises in dual-channel presale strategies by studying in depth the influence of channel selection on presale entities.

Based on the above results, this paper studies the impact of channel preference and product valuation differences on the presale decisions for innovative products and corporate profits. This study fills the limitation of existing literatures, which has focused only on a single variable, and provides more comprehensive theoretical support for the formulation of presale strategies. Through the comprehensive analysis of multiple variables, the research results are more closely related to actual production and operation. Moreover, this study also fills a research gap in the area of presale entity selection and provides theoretical guidance for enterprises. This approach can help enterprises better grasp market demand and improve the sales effect of products. Table 1 summarizes the main distinctions between this study and the most related literature.

**Table 1:** Distinctions between this study and the most related literature.

Literature	Innovative Product	valuation difference	channel	Presale entity
Wu et al. (2021) [29]	√	√	single	R
Xu et al. (2021) [37]	×	×	single	R
Du et al. (2022) [30]	√	√	single	R
Guo et al. (2022) [38]	×	×	single	R
Zhang et al. (2023) [31]	√	√	dual	R
Wang et al. (2023) [41]	√	×	single	M
Zhao 和 Li (2023) [39]	×	×	dual	M
Wu et al. (2023) [40]	×	×	dual	R
This paper	√	√	dual	M or R

The innovations of this paper are as follows: (1) Comparing the distinctions in decision-making and profit between the no-presale and presale decisions of different entities and exploring the impacts of channel preferences and product valuation differences on presale decision-making and innovative product sales. (2) Considering the impact of channel preference on the selection of presale entities and equilibrium results, the conditions of optimal decision-making under different channel preferences are given. (3) It analyzes the impact of consumers’ valuation differences related to innovative products on enterprises’ presale decisions and profits and discusses the presale strategy of enterprises in the face of different degrees of valuation difference. Through theoretical and numerical analysis, the managerial insights obtained in this paper can provide theoretical support and decision-making guidance for participants.

## 3. Problem description and basic assumptions

### 3.1 Problem description

**Fig 1:**
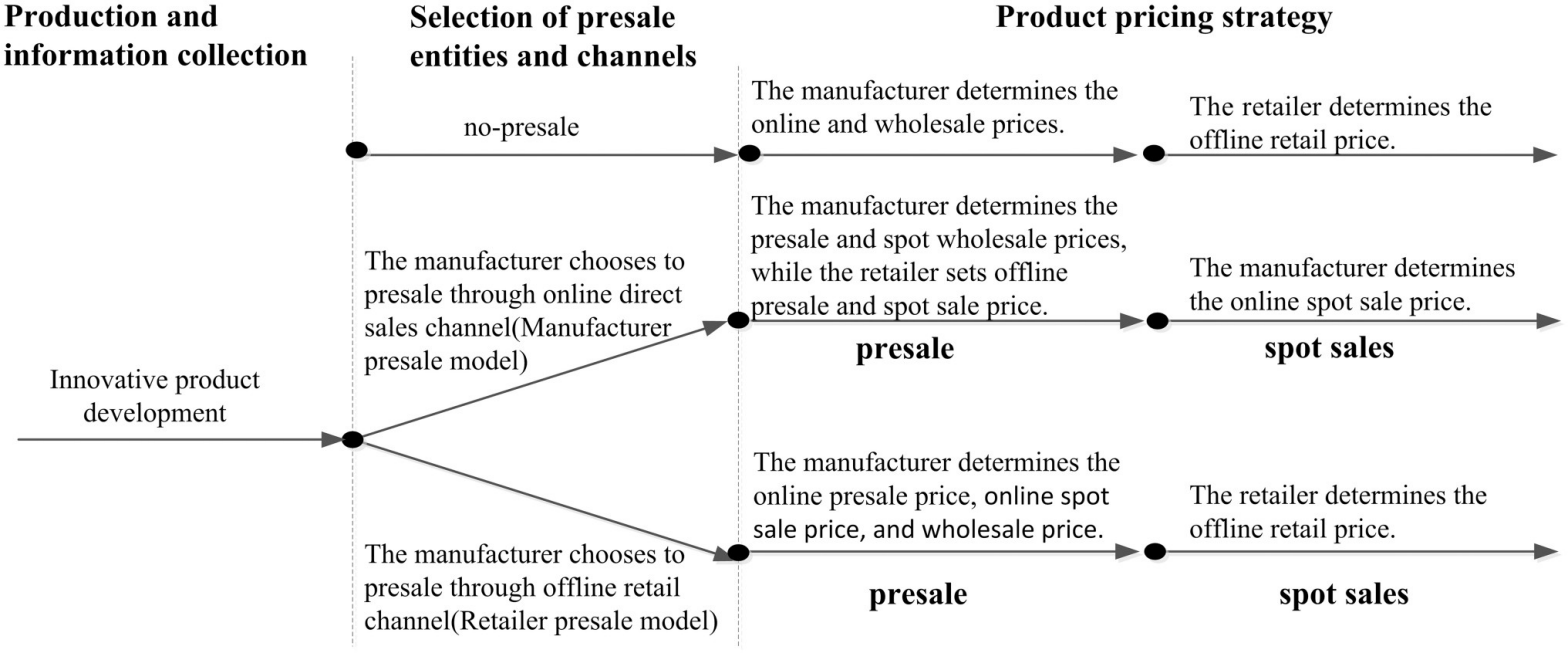
Timeline of the game process.

In this paper, we consider a monopolistic manufacturer selling the same product to strategic consumers through online and offline channels. The sales process is carried out in two stages: presale and spot sale. During the presale period, enterprises release the presale and spot sale prices, and consumers choose the time to buy the product by comparing their utility. The spot sale period comes after the presale period. The spot sale enterprise sets the spot price, and consumers compare the utility of buying products from different channels and determine how to purchase them. In this paper, the superscripts *i* = *N*, *R*, *M* are used to denote three models: the no-presale model, the retailer presale model, and the manufacturer presale model. In the dual-channel system, the manufacturer is dominant, and the retailer is the follower. They are both completely rational “economic men”. According to previous studies [26, 35, 39, 42], the timeline of the game process is shown in Fig 1.

### 3.2 Basic assumptions

Based on the above situation, the following assumptions are made: (1) All consumers are strategic and arrive in the market before the presale begins, and each consumer buys at most one product. (2) Consumers’ perceived value of innovative products (denoted by *v*) follows a uniform distribution in the range [0, 1]. According to the literature [20, 27, 35, 38, 39, 41], the consumer’s utility from buying products is $\mu_{xy}^{i}=\theta_{y}v-p_{xy}^{i}$ in the presale period and $\mu_{xy}^{i}=\theta_{y}\delta v-p_{xy}^{i}$ in the spot sale period. Here, *θ*_*y*_ represents the channel preference of the consumer. We assume that the offline channel preference (denoted by *θ*_2_) is 1. Due to the inability to directly contact and experience products online, consumers have less preference for the online channel than for the offline channel. Since there is some substitutability between channels, the consumer’s preference for the online direct channel (denoted by *θ*_1_) is *θ* and *θ* ∈ (0.5,1). When *θ* is larger, consumers’ acceptance of the direct channel is greater. *δ* denotes the valuation discount for consumers who delay purchasing products. The higher the out-of-stock rate of the product and the greater the consumer’s pursuit of innovative technology are, the lower the valuation discount. (3) The production cost is zero.

For convenience, we summarize the notations used in this paper in Table 2.

**Table 2:** Notations and descriptions for the model.

Notations	Descriptions
**x**	x = {a, s} represent presale and spot sale, respectively
**y**	y = {1, 2} represent the online direct sales channel and offline retail channel, respectively
**ν**	Consumer perceived value of an innovative product
**μ**	Consumer utility obtained by purchasing a product
**θ**	Consumer’s channel preference
**δ**	Product valuation discount
***w*_*xy*_**	Wholesale price per unit product
**c**	The marginal cost of presale per unit product, which also indicates the effort made by the presale entity
***p*_*xy*_**	Sales price per unit product
**π_*m*_**	Manufacturer’s profit
**π_*r*_**	Retailer’s profit
**π_*sc*_**	Supply chain’s profit

## 4. Modeling and analysis

### 4.1 No-presale model

In the no-presale model, the manufacturer adopts a dual-channel approach to sell products. The consumer utility is $\mu_{s1}^{N}=\delta \theta v-p_{s1}^{N}$ for the direct channel, and $\mu_{s2}^{N}=\delta v-p_{s2}^{N}$ for the retail channel. When $p_{s2}^{N}>p_{s1}^{N}$, the demands of both channels are positive. When $p_{s2}^{N}>p_{s1}^{N}$, the demand in the direct channel is zero. Therefore, only the case of $p_{s2}^{N}>p_{s1}^{N}$ is considered in this paper. Letting $\mu_{s2}^{N}=\mu_{s1}^{N}$, the critical value can be obtained $v=\left(p_{s2}^{N}-p_{s1}^{N}\right)/\left[\left(1-\theta \right)\delta \right]$. When $\mu_{s1}^{N}\geq 0$ and $\mu_{s1}^{N}\geq \mu_{s2}^{N}$, the consumers choose the direct channel and their willingness to purchase is $p_{s1}^{N}/\left(\theta \delta \right)\leq v\leq \left(p_{s2}^{N}-p_{s1}^{N}\right)/\left[\left(1-\theta \right)\delta \right]$. Therefore, the demand function of the direct channel is $q_{s1}^{N}=\int_{p_{s1}^{N}/\left(\theta \delta \right)}^{\left(p_{s2}^{N}-p_{s1}^{N}\right)/\left[\left(1-\theta \right)\delta \right]}f\left(v\right)dv=\left(p_{s2}^{N}-p_{s1}^{N}\right)/\left[\left(1-\theta \right)\delta \right]-p_{s1}^{N}/\left(\theta \delta \right)$. When $\mu_{s2}^{N}\geq 0$ and $\mu_{s2}^{N}\geq \mu_{s1}^{N}$, the consumers choose the retail channel; here, $\left(p_{s2}^{N}-p_{s1}^{N}\right)/\left[\left(1-\theta \right)\delta \right]\leq v\leq 1$, so the demand function of the retail channel is $q_{s2}^{N}=\int_{\left(p_{s2}^{N}-p_{s1}^{N}\right)/\left[\left(1-\theta \right)\delta \right]}^{1}f\left(v\right)dv=1-\left(p_{s2}^{N}-p_{s1}^{N}\right)/\left[\left(1-\theta \right)\delta \right]$.

The profit functions of the manufacturer and retailer are formulated as follows:

$$\text{max}\;\pi_{m}^{N}\left(p_{s1}^{N},w_{s2}^{N}\right)=p_{s1}^{N}\cdot q_{s1}^{N}+w_{s2}^{N}\cdot q_{s2}^{N}$$ (1)

$$\text{max}\;\pi_{r}^{N}\left(p_{s2}^{N}\right)=\left(p_{s2}^{N}-w_{s2}^{N}\right)\cdot q_{s2}^{N}$$ (2)

**Lemma 1**. In the no-presale model, the optimal wholesale price is $w_{s2}^{N*}=\delta /2$, the direct sale price is $p_{s1}^{N*}=\theta \delta /2$, and the retail price is $p_{s2}^{N*}=\left(3-\theta \right)\delta /4$, the manufacturer’s profit is $\pi_{m}^{N*}=\left(1+\theta \right)\delta /8$, and the retailer’s profit is $\pi_{R}^{N*}=\left(1-\theta \right)\delta /16$.

In the no-presale model, the offline channel brings higher product experience value to consumers. Therefore, under the same price, the utility obtained through offline purchases is greater than that obtained through online purchases, at which point consumers prefer the offline channel. When the online and offline utilities are consistent, the price of the offline product is higher than that of the online product.

Channel preference does not affect the wholesale price; as the difference in consumer channel preference increases, that is, *θ* decreases, consumers are more willing to choose the retail channel, so the manufacturer’s profit decreases and the retailer’s profit increases. In contrast, as channel differentiation narrows, the gap in product experience value between different channels narrows. At this point, the online price increases, while the offline price decreases, and the retailer’s marginal profit per unit of product is smaller than that of the manufacturer.

### 4.2 Retailer presale model

In the retailer presale model, the consumer utility can be described as $\mu_{a2}^{R}=v-p_{a2}^{R}$ for the retailer channel during the presale period, $\mu_{s2}^{R}=\delta v-p_{s2}^{R}$ for the retailer channel during the spot sale period, $\mu_{s1}^{R}=\theta \delta v-p_{s1}^{R}$ for the direct channel during the spot sale period.

In the presale period, consumers make decisions to maximizing their utility. Let us assume that $\mu_{a2}^{R}=\mu_{s2}^{R}$, and we can obtain the critical value for purchase intentions in the presale period $v=\left(p_{a2}^{R}-p_{s2}^{R}\right)/\left(1-\delta \right)$. When $\mu_{a2}^{R}\geq 0$ and $\mu_{a2}^{R}\geq \mu_{s2}^{R}$, consumers choose to purchase in the presale period, and the presale demand function of the retail channel is $q_{a2}^{R}=\int_{\left(p_{a2}^{R}-p_{s2}^{R}\right)/\left(1-\delta \right)}^{1}f\left(v\right)dv=1-\left(p_{a2}^{R}-p_{s2}^{R}\right)/\left(1-\delta \right)$. Otherwise, consumers choose to wait. During the spot sale period, let us assume that $\mu_{s1}^{R}=\mu_{s2}^{R}$, and we obtain the critical value for $v=\left(p_{s2}^{R}-p_{s1}^{R}\right)/\left[\left(1-\theta \right)\delta \right]$. When $\mu_{s1}^{R}\geq 0$ and $\mu_{s1}^{R}\geq \mu_{s2}^{R}$, consumers choose to purchase in the direct channel; here, consumers’ demand function can be described as $q_{s1}^{R}=\int_{p_{s1}^{R}/\left(\theta \delta \right)}^{\left(p_{s2}^{R}-p_{s1}^{R}\right)/\left[\left(1-\theta \right)\delta \right]}f\left(v\right)dv=\left(p_{s2}^{R}-p_{s1}^{R}\right)/\left[\left(1-\theta \right)\delta \right]-p_{s1}^{R}/\left(\theta \delta \right)$. When $\mu_{s2}^{R}\geq 0$ and $\mu_{s2}^{R}\geq \mu_{s1}^{R}$, consumers choose to purchase in the retailer channel, and in this situation, the consumer demand function is $q_{s2}^{R}=\int_{\left(p_{s2}^{R}-p_{s1}^{R}\right)/\left[\left(1-\theta \right)\delta \right]}^{\left(p_{a2}^{R}-p_{s2}^{R}\right)/\left(1-\delta \right)}f\left(v\right)dv=\left(p_{a2}^{R}-p_{s2}^{R}\right)/\left(1-\delta \right)-\left(p_{s2}^{R}-p_{s1}^{R}\right)/\left[\left(1-\theta \right)\delta \right]$; otherwise, they choose to leave.

The profit functions of the manufacturer and retailer can be formulated as follows:

$$\text{max}\;\pi_{m}^{R}\left(w_{a2}^{R},w_{s2}^{R},p_{s1}^{R}\right)=w_{a2}^{R}\cdot q_{a2}^{R}+w_{s2}^{R}\cdot q_{s2}^{R}+p_{s1}^{R}\cdot q_{s1}^{R}$$ (3)

$$\text{max}\;\pi_{r}^{R}\left(p_{a2}^{R},p_{s2}^{R}\right)=\left(p_{a2}^{R}-w_{a2}^{R}-c\right)\cdot q_{a2}^{R}+\left(p_{s2}^{R}-w_{s2}^{R}\right)\cdot q_{s2}^{R}$$ (4)

**Lemma 2**. In the retailer presale model, the optimal wholesale price is $w_{a2}^{R*}=\left(1-c\right)/2$, $w_{s2}^{R*}=\delta /2$, the direct presale price is $p_{a2}^{R*}=\left(c-\delta \theta +3\right)/4$, the spot sale price is $p_{s1}^{R*}=\theta \delta /2$, the retailer spot sale price is $p_{s2}^{R*}=\left(3-\theta \right)\delta /4$, the manufacturer’s profit is $\pi_{m}^{R*}=\left[{\left(1-c\right)}^{2}+\left(2c+\theta -1\right)\delta \right.\left.-\theta \delta^{2}\right]/\left[8\left(1-\delta \right)\right]$, and the retailer’s profit is $\pi_{r}^{R*}=\left[{\left(1-c\right)}^{2}-\left(1-2c+\theta \right)\delta +\theta \delta^{2}\right]/\left[16\left(1-\delta \right)\right]$.

**Proposition 1**. (1) $p_{a2}^{R*}>p_{s2}^{R*}>p_{s1}^{R*}$; (2) when $0\leq c\leq \left(1-\delta \right)/2$, $q_{s1}^{R*}\geq q_{a2}^{R*}\geq q_{s2}^{R*}$; and when $\left(1-\delta \right)/2<c\leq 1-\delta$, $q_{s1}^{R*}\geq q_{s2}^{R*}\geq q_{a2}^{R*}$.

Proposition 1 indicates that in the retailer presale model, to encourage consumers to purchase products during the presale period and reduce uncertainty in the valuation of innovative products, the retailer needs to exert more effort. Therefore, the total cost of the presale period is greater than that of the spot sale period, and tends to be greater when pricing the product. Consumers can obtain products in advance through presale to meet their inherent demand for innovative products. Therefore, they are willing to pay more for presales. Furthermore, due to the loss of part of the opportunity cost of delaying the purchase of the product, resulting in a lower product valuation of the spot sales than of presales. To ensure that the spot sale volume is more than zero, the spot sale price should be lower than the presale price. In the spot sale period, consumers’ preference for direct channels decreases due to the inability to contact and experience products, so they are more inclined to purchase products through retailer channels. To promote online sales, the manufacturer adopts the low-price strategy to attract consumers, thus the spot sale price of direct sales is lower than the retail price, and online sales are greater than offline sales. However, regardless of the incentive measures adopted, the direct sales and retail sales of the product are equal.

To motivate consumers to purchase products, the retailer exerts different amounts of effort and generates different presale costs, which affects the distribution of sales between channels. When the presale cost is high, the unit profit obtained from the presale decreases, and the retailer will reduce the presale volume and sell products in the form of spot sales. When the presale cost is low, the retailer increases the presale volume and sells products in presale. Regardless of how the presale cost changes, the total sales of offline remain the same.

### 4.3 Manufacturer presale model

In the manufacturer presale model, the consumer utility from direct channels in the presale stage can be described as: $\mu_{a1}^{M}=\theta v-p_{a1}^{M}$; for retail channel in the spot sale stage, consumer utility is $\mu_{s2}^{M}=\delta v-p_{s2}^{M}$; and for direct channel in the spot sale stage, consumer utility is $\mu_{s1}^{M}=\theta \delta v-p_{s1}^{M}$. The demand functions are as follows:

$$\left\{\begin{array}{c} q_{a1}^{M}=\int_{\frac{p_{a1}^{M}-p_{s1}^{M}}{\theta \left(1-\delta \right)}}^{1}f\left(v\right)dv=1-\frac{p_{a1}^{M}-p_{s1}^{M}}{\theta \left(1-\delta \right)}\\ q_{s1}^{M}=\int_{\frac{p_{s2}^{M}-p_{s1}^{M}}{\left(1-\theta \right)\delta }}^{\frac{p_{a1}^{M}-p_{s1}^{M}}{\theta \left(1-\delta \right)}}f\left(v\right)dv=\frac{p_{a1}^{M}-p_{s1}^{M}}{\theta \left(1-\delta \right)}-\frac{p_{s2}^{M}-p_{s1}^{M}}{\left(1-\theta \right)\delta }\\ q_{s2}^{M}=\int_{\frac{p_{s1}^{M}}{\theta \delta }}^{\frac{p_{s2}^{M}-p_{s1}^{M}}{\left(1-\theta \right)\delta }}f\left(v\right)dv=\frac{p_{s2}^{M}-p_{s1}^{M}}{\left(1-\theta \right)\delta }-\frac{p_{s1}^{M}}{\theta \delta } \end{array}\right.$$

The profit functions of the manufacturer and retailer can be formulated as follows:

$$\text{max}\;\pi_{m}^{M}\left(p_{a1}^{M},p_{s1}^{M},w_{s2}^{M}\right)=\left(P_{a1}^{M}-c\right)\cdot q_{a1}^{M}+p_{s1}^{M}\cdot q_{s1}^{M}+w_{s2}^{M}\cdot q_{s2}^{M}$$ (5)

$$\text{max}\;\pi_{r}^{M}\left(p_{s2}^{M}\right)=\left(p_{s2}^{M}-w_{s2}^{M}\right)\cdot q_{s2}^{M}$$ (6)

**Lemma 3**. In the manufacturer presale model, the optimal wholesale price of products is $w_{s2}^{S*}=\left[\left(4-3\delta \right)\theta^{2}+\left(4-4c-5\delta \right)\theta +4c\right]\delta /A$, the presale price in direct channel is $p_{a1}^{S*}=\left[8\left(c+\theta \right)\left(1-\delta \right)\right.-\left.\left(1-\theta \right)\delta^{2}\right]\theta /A$, the spot sale price is $p_{s1}^{S*}=\theta \delta /2$, the retailer price is $p_{s2}^{M*}=\left[\left(2-\delta \right)\theta^{2}+\right.\left.\left(6-6c-7\delta \right)\theta +6c\right]\delta /A$, the profit of the manufacturer is $\pi_{m}^{S*}=\left[\left(8+\delta^{2}-8\delta \right)\theta^{2}+\right.\left(12\delta c-\delta^{2}-16c\right)\theta +8c^{2}\left.+4\delta c\right]/\left(2A\right)$, and the profit of the retailer is $\pi_{r}^{S*}=4\left(1-\theta \right){\left[\left(\theta \left(1-\delta \right)+c\right)\right]}^{2}\delta /A^{2}$, where $A=2\left[8\theta -\left(1+7\theta \right)\delta \right]$.

**Proposition 2** (1) $p_{s2}^{M*}>p_{a1}^{M*}>p_{s1}^{M*}$; (2) when $c_{2}<c<c_{1}$, $q_{a1}^{M*}<q_{s1}^{M*}<q_{s2}^{M*}$; when $c_{3}<c<c_{2}$,$q_{s1}^{M*}<q_{a1}^{M*}<q_{s2}^{M*}$; and when $0<c<c_{3}$, $q_{s1}^{M*}<q_{s2}^{M*}<q_{a1}^{M*}$, where $c_{1}=\left(4\theta -\left(3\theta +1\right)\delta \right)/4$, $c_{2}=\left(10\theta -\left(7\theta +3\right)\delta \right)/14$, $c_{3}=\left[3\theta -\left(2\theta +1\right)\delta \right]/5$.

Proposition 2 indicates that due to differences in channel preference, consumers have a greater valuation of offline products in the spot sale. When consumer utility is the same between the online and offline channels, the price of offline products is greater. When the prices of online and offline products are consistent, consumers are more willing to purchase products through retail channel; thus, the offline product sales are higher than online. Although innovative products have certain appeal to consumers, not all consumers participate in the presale. Due to the impact of presale costs, consumers may choose to wait or leave when facing higher presale prices. When the presale cost is less than *c*_3_, the presale profit of the unit product is greater than the spot sale profit. The manufacturer increases the presale volume to increase profits. When the presale cost is greater than *c*_1_, the manufacturer is not profitable due to the high presale cost, so the presale will not be carried out. All products are sold through spot sales, and the direct sales volume is equal to the retail sales volume. When the presale cost is between *c*_1_ and *c*_3_, the manufacturer allocates the sales reasonably by comparing the presale profit and spot sales profit per unit product, and the direct sales volume is greater than the retailer sales volume. When the presale cost is high, the manufacturer’s direct sales profit is lower than the retail profit. Therefore, the manufacturer will choose the retail spot sales model as the main option, supplemented by the online spot sale model, which has the lowest presale volume.

### 4.4 Comparison of model results

**Proposition 3**. In the different presale models, the influence of channel preferences on supply chain equilibrium decisions is as follows:

(1) $\frac{\partial p_{a2}^{R*}}{\partial \theta }<0$, $\frac{\partial p_{s2}^{R*}}{\partial \theta }<0$, $\frac{\partial p_{s1}^{R*}}{\partial \theta }>0$, $\frac{\partial \pi_{m}^{R*}}{\partial \theta }>0$, $\frac{\partial \pi_{r}^{R*}}{\partial \theta }<0$;
(2) $\frac{\partial p_{a1}^{M*}}{\partial \theta }>0$, $\frac{\partial p_{s1}^{M*}}{\partial \theta }>0$, $\frac{\partial q_{a1}^{M*}}{\partial \theta }>0$, $\frac{\partial q_{s2}^{R*}}{\partial \theta }<0$, $\frac{\partial q_{s1}^{R*}}{\partial \theta }<0$, when $\frac{1}{2}<\theta <\bar{\theta }$, $\frac{\partial p_{s2}^{M*}}{\partial \theta }<0$; however, when $\bar{\theta }<\theta <1$, $\frac{\partial p_{s2}^{M*}}{\partial \theta }>0$, where $\bar{\theta }=\frac{\left(2-\delta \right)+4\sqrt{3\left(1-\delta \right)\left(2-\delta \right)\left(8c+\delta -7c\delta -\delta^{2}\right)}}{\left(2-\delta \right)\left(8-7\delta \right)}$.

Proposition 3 shows that in the retailer presale model, as the difference in channel preferences narrows, i.e., *θ* increases, consumers’ valuation of online products increases, which means that when consumers purchase online products, their utility will also increase correspondingly. Therefore, consumers are willing to purchase these products at higher prices. In the manufacturer presale model, as *θ* increases, consumers’ acceptance of online products improves, leading to a jump in presale prices and sales volume. When *θ* exceeds a certain threshold, the manufacturer narrows the profit gap per unit of product between channels by increasing wholesale prices, leading to a decrease in retailer profits. To slow the decline in presales, the retailer has to lower prices to retrieve lost consumers. In this case, the manufacturer should adopt the direct presale model to attract more consumers, which can not only increase their profits and market share, but also alleviate the pressure on retailers when their profit is reduced. However, as *θ* shrinks, consumers are more willing to purchase products through the offline channel, and the manufacturer’s unit product profit under the retail presale model gradually becomes greater than that under the direct presale model. Under these circumstances, the manufacturer should choose to collaborate with the retailer to carry out presale activities. In this way, the profit level of the manufacturer can be guaranteed, and the interests of the retailer can be maintained to achieve a win-win situation.

**Proposition 4**. In the different presale models, the product valuation discount on supply chain equilibrium decisions is as follows:

(1) $\frac{\partial p_{a2}^{R*}}{\partial \delta }<0$, $\frac{\partial p_{s2}^{R*}}{\partial \delta }>0$, $\frac{\partial p_{s1}^{R*}}{\partial \delta }>0$, $\frac{\partial q_{a2}^{R*}}{\partial \delta }<0$, $\frac{\partial q_{s2}^{R*}}{\partial \delta }>0$, $\frac{\partial q_{s1}^{R*}}{\partial \delta }=0$;
(2) $\frac{\partial p_{a1}^{M*}}{\partial \delta }>0$, $\frac{\partial p_{s1}^{M*}}{\partial \delta }>0$, $\frac{\partial p_{s2}^{M*}}{\partial \delta }>0$, $\frac{\partial q_{a1}^{M*}}{\partial \delta }<0$, $\frac{\partial q_{s2}^{R*}}{\partial \delta }>0$, $\frac{\partial q_{s1}^{R*}}{\partial \delta }>0$.

Proposition 4 indicates that in the retailer presale model, there is a positive correlation between the spot sale price of the product and the product valuation discount, while the relationship between the presale price and the product valuation discount is negative. Thus, the higher the valuation discount of innovative products, the more unfavorable the product is for presale. In the manufacturer presale model, the price of the product increases as *θ* increases. When *θ* is lower, the difference in product valuation between spot sales and presales is large, and consumers prefer the presale model. In this situation, the manufacturer should choose the retailer presale model to expand presale profits by increasing presale prices. However, owing to the decrease in spot sale prices and quantities, the decrease in spot sale profits exceeds the increase in presale profits, resulting in a decrease in retailer profits. A higher *θ* indicates that the consumers are more patient and are more likely to delay their purchase, and in turn presale prices are lower and spot sales are higher. At this point, consumers’ valuation of spot products increases, and their acceptance of spot sale prices also increases, which also has a positive impact on presale prices. Consequently, the presale price increases. Accordingly, it is more suitable to adopt the retailer presale model. However, when the discount on product valuation exceeds a certain threshold, the increase in spot sale profits is greater than the decrease in presale profits, and the presale model is not recommended.

**Proposition 5**. Regardless of whether the manufacturer conducts presales and what kind of channel strategy they will adopt, the total sales volume of the product will not change.

Proposition 5 indicates that in an oligopoly market, the presale does not help companies increase their total sales of products; it only encourages consumers to make purchase decisions in advance. For consumers without a desire to purchase, presale strategies do not motivate them to purchase products. The purpose of enterprises implementing presales is to lock in consumers in advance and avoid significant fluctuations in demand due to changes in the demand market and competitors’ marketing strategies. Presale can help companies to produce with known demand, reduce inventory, and reduce risk.

**Proposition 6**. Comparative analysis of equilibrium decisions under three models:

(1) $p_{s1}^{N*}=p_{s1}^{R*}=p_{s1}^{M*}$, $p_{s2}^{N*}=p_{s2}^{R*}>p_{s2}^{M*}$, $p_{a2}^{R*}>p_{a1}^{M*}$;
(2) $q_{s1}^{M*}>q_{s1}^{R*}=q_{s1}^{N*}$, $q_{s2}^{N*}>q_{s2}^{M*}>q_{s2}^{R*}$, $q_{a2}^{R*}>q_{a1}^{M*}$.

Proposition 6 clarifies that whether the manufacturer chooses to presale and the selection of the presale entity do not affect the spot sale prices of online products. In the retailer presale model, the main hope is that presales can bring them more benefits. To reduce the uncertainty of consumer valuation of innovative products, the retailer needs to make more efforts to motivate consumers, so the total cost of the presale period is higher than that of the spot sale period. Therefore, the price of presales is often higher than that of in the spot sale period. However, the purpose of the manufacturer’s presale is to make profits and expand market share so that when the manufacturer conducts presale, the presale price is usually lower than the spot sale price. Although innovative products have certain appeal to consumers, not all consumers will participate in the presale. For innovative and highly timely products, consumers are willing to pay more to obtain the product in advance. The presale not only meets their inherent need for innovative products but also allows them to become the first batch of users in the early stages when products launch, thereby obtaining a unique sense of satisfaction. In this instance, the manufacturer should choose the retailer presale model. When facing higher presale prices, consumers may also hesitate, and the manufacturer needs to use direct presale methods to retain consumers at low prices. Nonetheless, when the presale cost exceeds a certain threshold, the benefits brought about by presales are less than those brought about by spot sales. In this situation, the presale entity sells products only through the spot sales.

## 5. Numerical analysis

To further analyze the influence of channel preference and product valuation discounts on decision variables and supply chain profits. The MATLAB software was used to perform the numerical simulations (*θ* = 0.8, *δ* = 0.6, *c* = 0.1) and sensitivity analysis. The parameter values are based on several previous studies [22, 24, 26, 28] and further standardized as benchmarks.

### 5.1 The effect of channel preference on decision variables and profits

**Fig 2:**
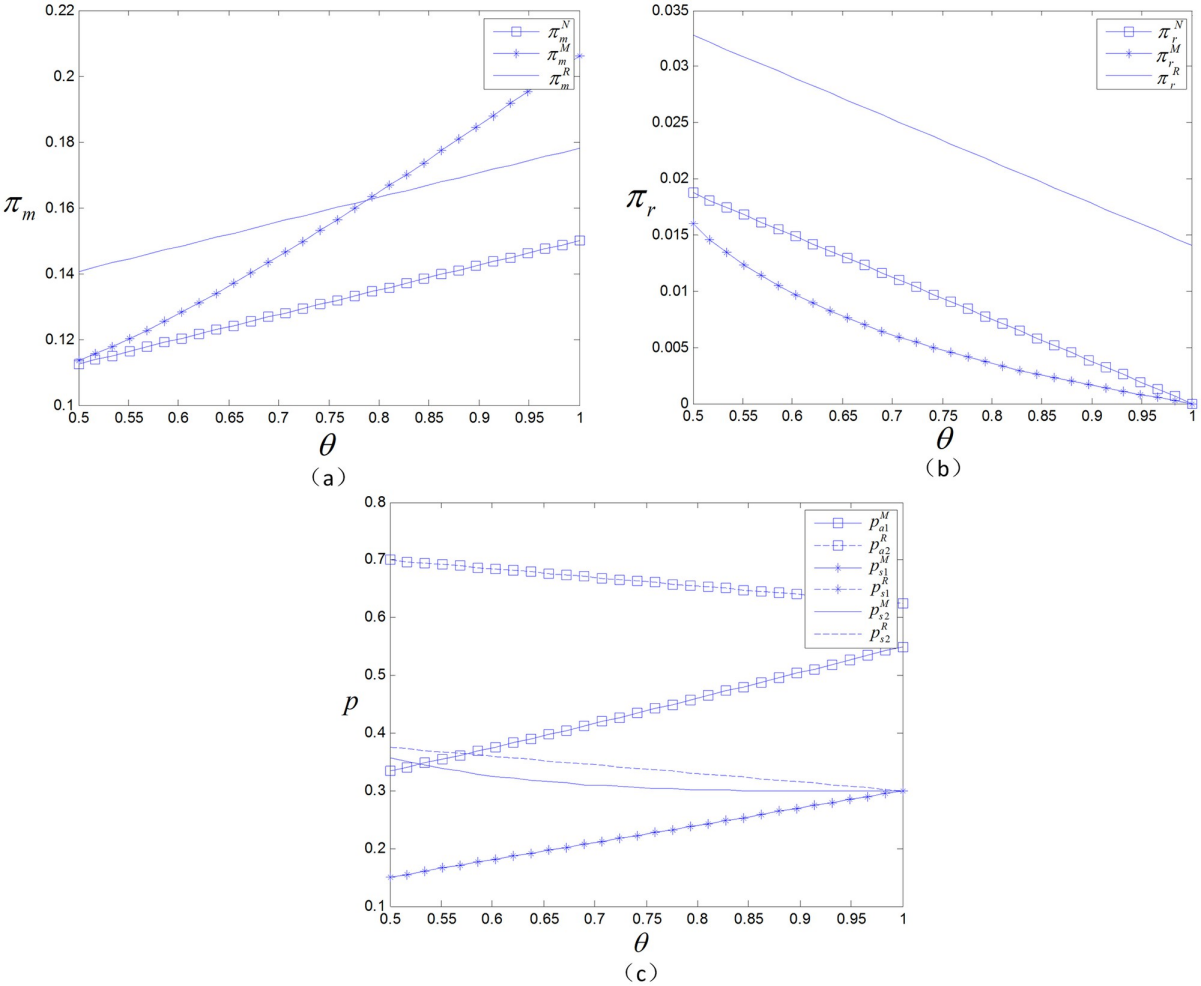
The effect of *θ* on prices and profits.

As shown in Fig 2, in the manufacturer presale model, the profits of the manufacturer and retailer are significantly affected by the channel preference. Specifically, the profit of the manufacturer will gradually increase as the difference in channel preferences narrows, and the speed of increase is faster than that under the retailer presale model. Compared with the no-presale case, presale can help manufacturers gain more profit, especially for innovative products. When the channel preference difference is small, i.e., *θ* is large, the manufacturer can achieve the maximum profit by choosing the direct presale model. However, as *θ* narrows, the manufacturer’s profit under the retailer presale model gradually becomes greater than that under the manufacturer presale model. In this case, the manufacturer should choose to collaborate with the retailer to carry out presale activities. In the no-presale model and the retailer presale model, the manufacturer’s profit increases as *θ* increases.

For the retailer, the choice of sales model has a significant impact on revenue. The retailer has the highest revenue in the retailer presale model, followed by the no-presale model, and has the minimum profit in the manufacturer presale model. The retailer’s profit decreases as *θ* increases; under these conditions, as the difference in channel preference narrows, the impact of different channel choices on profits decreases. Moreover, due to the lower pricing of direct channels, consumers are more willing to choose direct channels, resulting in fewer offline product sales, which reduces the profit of retailers.

**Fig 3:**
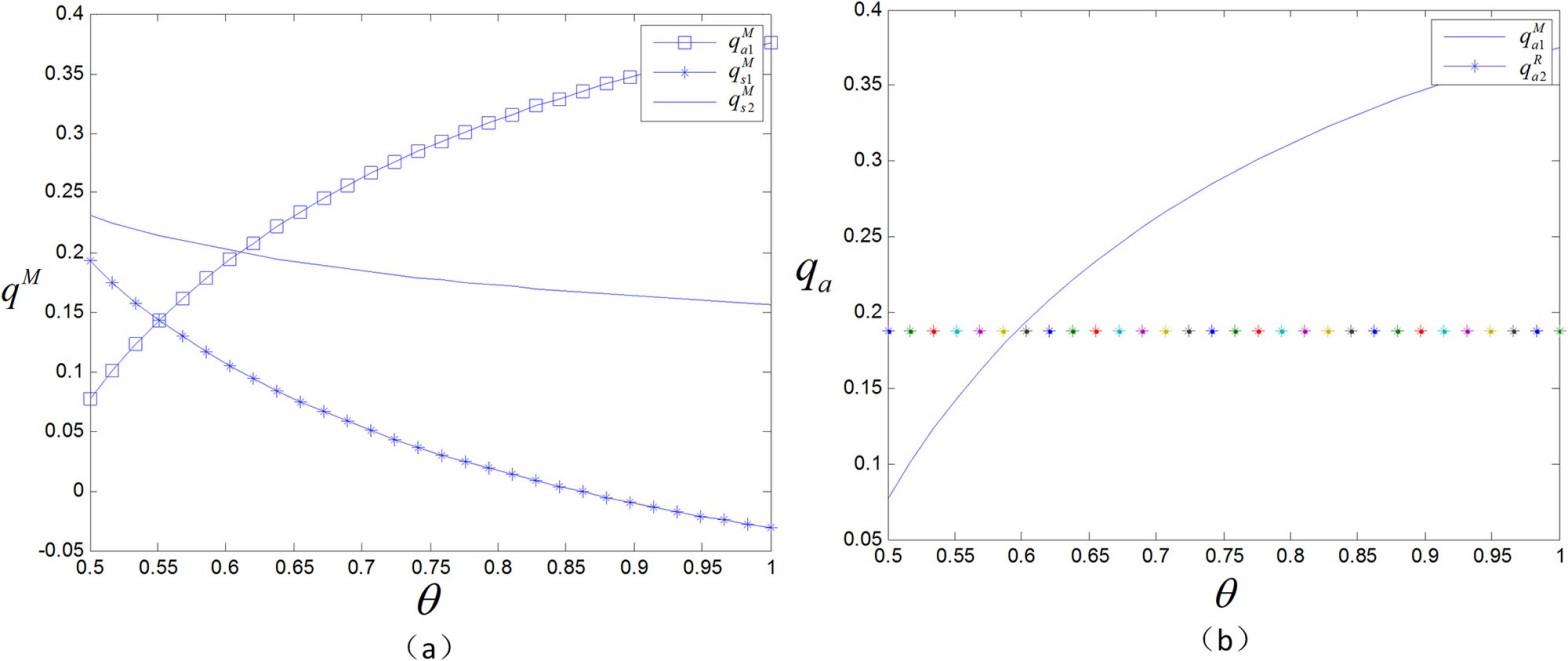
The effect of *θ* on sales.

From Fig 3, it can be seen that there is a positive correlation between the presale volume and consumers’ acceptance of the online channel in the manufacturer presale model. With the increasing acceptance of online shopping by consumers, demand is also growing. The retailer presale model has greater advantages in increasing presale volume. The value generated by the presale of different products varies; for example, fresh agricultural products can reduce the consumption of products in the circulation process and reduce the loss of freshness through presale. Therefore, the revenue from the presale of fresh agricultural products is far greater than that from spot sale.

Under these conditions, choosing the retailer presale model can effectively improve the presale volume of products, thereby increasing the profits of the entire supply chain. In addition, the retailer presale model can also provide other benefits. First, retailers can better understand consumer needs and preferences through presales to adjust the supply and inventory management strategies of products. Second, presales can help retailers obtain funds in advance, reduce the pressure of capital turnover, and improve the efficiency of capital utilization. Finally, presale can also provide retailers with better market competitive advantages and attract more consumers to choose presale products. However, it should be noted that retailers need to consider market demand, product characteristics, and supply chain management capabilities when choosing presale models. Only rational use of the presale can maximize the amount of presale products needed to optimize the supply chain and increase profits.

### 5.2 The effect of the product valuation discount on decision variables and profits

**Fig 4:**
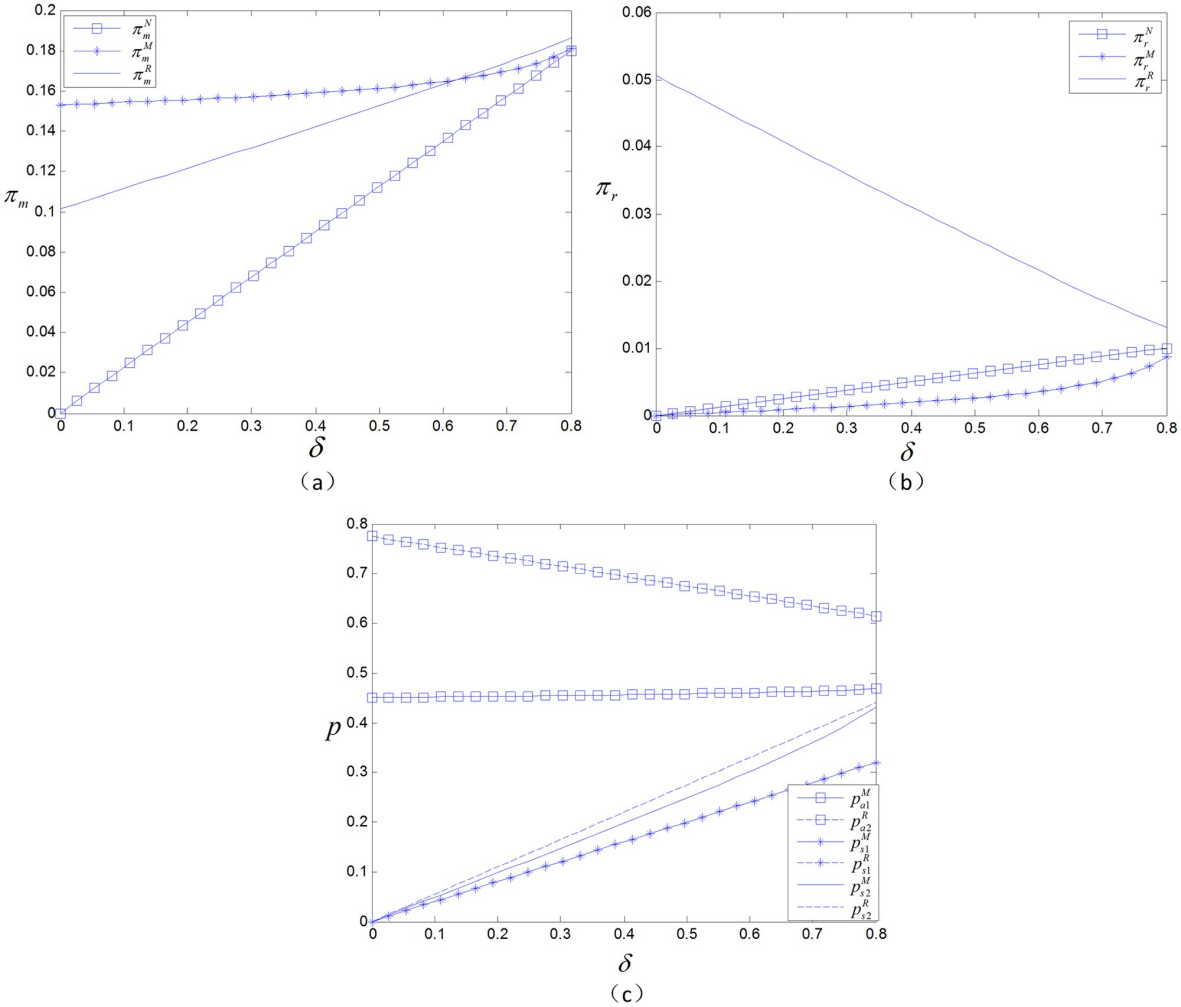
The effect of *δ* on prices and profits.

Fig 4 shows that in the case of a low product valuation discount, the consumer’s purchasing decisions depend mainly on the stage of the product and the corresponding price. In this case, the spot direct selling price is usually the lowest, so consumers are more inclined to choose the direct spot sale model, since they can immediately obtain the product without waiting for the presale period to end. However, as the product valuation discount gradually increases, the utility gap between presales and spot sales will narrow. This means that consumers will be more patient when purchasing products, willing to spend time and effort comparing the advantages and disadvantages of different products, and waiting for the best purchasing time and channel. Therefore, in this situation, the manufacturer should increase the presale price to induce consumers to choose the spot sale method. In this way, the retailer presale model will be more conducive to increasing the manufacturer’s profit. In contrast, when the product valuation discount is low, the manufacturer should lower the presale price to attract consumers. By doing so, consumers are more willing to participate in presale activities; therefore, the manufacturer’s profit grows. Under these circumstances, the profit obtained by the manufacturer through the presale is optimal.

As the product valuation discount increases, consumers are more inclined to purchase products via spot sale when presale is not offered or when the manufacturer presells. At this time, the retailer’s offline sales increase and its profits increase. However, in the retailer presale model, the decrease in the presale volume is greater than the increase in the spot sale volume, resulting in a decrease in total profit for the retailer. The profit of the manufacturer is much greater than that of the retailer, and the overall profit trend of the supply chain is similar to the profit curve of the manufacturer.

**Fig 5:**
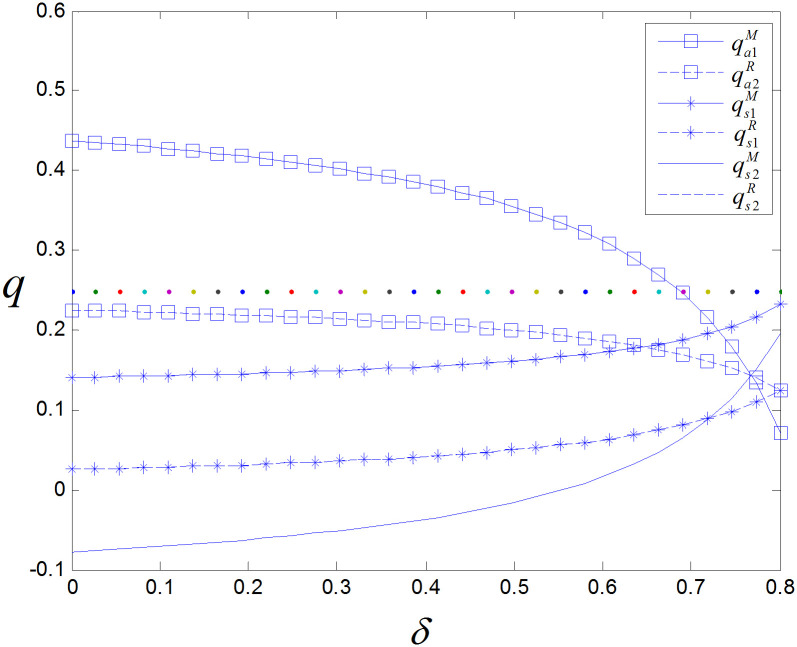
The effect of *δ* on sales.

From Fig 5, it can be seen that compared to the retailer presale model, the manufacturer’s presale price remains unchanged as *δ* increases. Due to the shift in consumer demand for spot sales, the spot sale volume increases, and the presale volume decreases. To reduce the loss caused by the loss of presale customers, the retailer must lower presale prices. In the manufacturer presale model, the presale volume also decreases, but the manufacturer compensates for the loss of presale profits by increasing the spot and wholesale prices of the product; thus, the presale price remains the same.

### 5.3 The effect of channel preference and product valuation discount on profits

**Fig 6:**
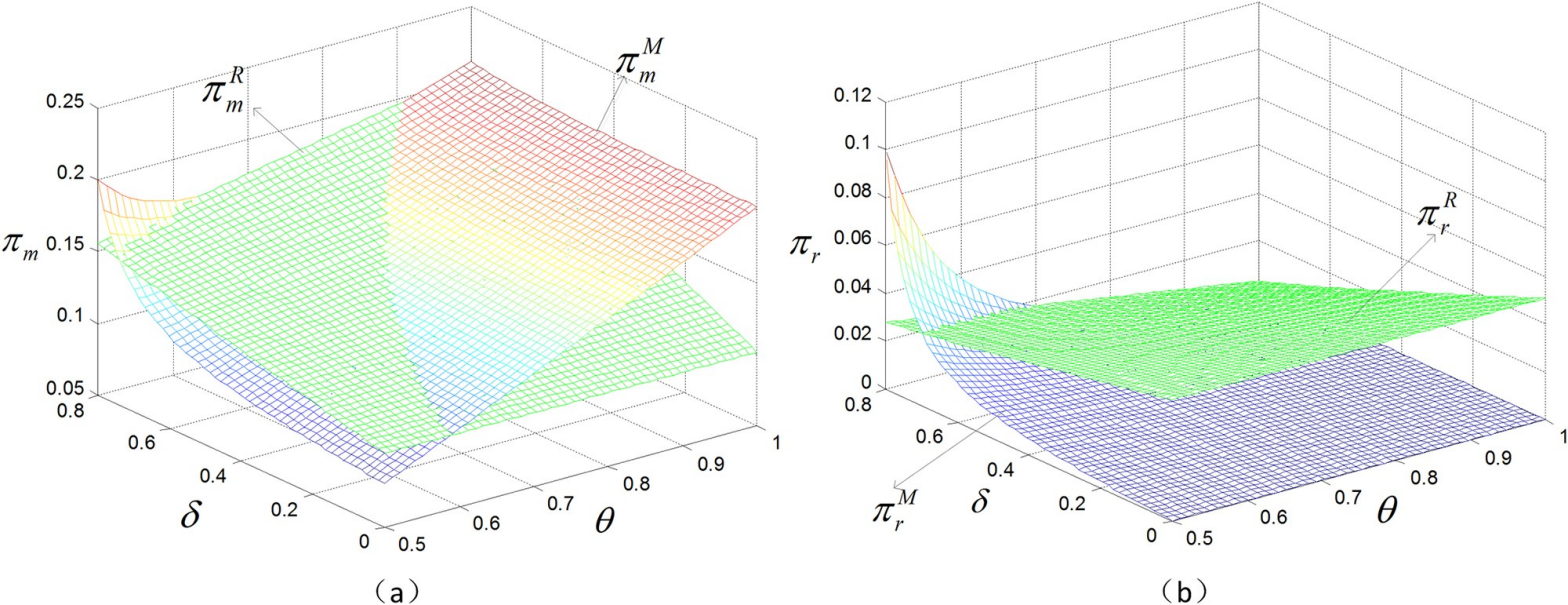
The effect of *θ* and *δ* on profits under two types of advance selling entities.

When *δ* and *θ* increase simultaneously, consumers who are not sensitive to channel differences and have a high valuation of spot products will purchase products through the spot sale channel. Due to the narrowing of the difference between channels, the wholesale price of products increases, and not only sales but also the marginal profit per unit of product will shrink for the retailer. At this point, the spot sale volume of online products increases, the manufacturer’s profit increases, and the retailer’s profit decreases. This is depicted in Fig 6.

In the retailer presale model, due to the increased utility brought about by spot sales, consumers choose to wait for spot sales during the presale period, thereby reducing the retailer’s presale profits. Moreover, as the wholesale price increases, the marginal profit per unit of product decreases. The retailer’s presale revenue and spot sale revenue both decrease, so the profit decline is greater than that in the manufacturer presale case. Therefore, when *δ* and *θ* simultaneously increase, the spot sale online model should be chosen. Similarly, when *δ* and *θ* both decrease, due to the increase in the difference between channels, consumers choose retail channels to reduce the risk of purchasing products. When the product valuation discount decreases, consumers are not willing to wait for spot sales. In this case, the manufacturer should choose the retailer presale model. When *δ* increases and *θ* decreases, and because of the increase in the product valuation discount, the difference in utility between presales and spot sales narrows, so consumers choose the spot sale channel. Moreover, as the channel difference increases, the utility generated by the online channel is far less than that generated by the offline channel. Under these conditions, the manufacturer chooses to purchase products through the spot sale offline model. When *δ* decreases and *θ* increases, the product valuation discount decreases and the channel difference narrows. Under these conditions, consumers can achieve greater utility by choosing the presale model. In the manufacturer presale model, the manufacturer raises the wholesale price to increase the marginal profit of a unit of product. Because the difference between channels is reduced, the sales volume of online products increases, so the manufacturer’s profit increases while the retailer’s profit decreases. However, the profit loss is greater in the retailer presale model, so the manufacturer presale model should be chosen.

## 6. Conclusion

### 6.1 Research conclusions

With the rapid development of technology and increasingly fierce market competition, the speed of product updates and iterations is also accelerating. This means that the lifecycle of products is becoming increasingly shorter, and manufacturers need to launch new products more quickly to meet consumer needs. To withstand fierce competition, manufacturers have begun to adopt presales to obtain consumer feedback and preference information. Presales can help manufacturers learn consumers’ expectations and demands for products in advance, reduce production costs and inventory risks, and improve the market competitiveness of products. However, in the literature, the impacts of channel preference and product valuation differences on product presale strategies have been widely studied separately. This paper combines channel preference and product valuation difference and jointly explores the influence of the two on the presale strategy of innovative products, aiming to fill this research gap. In practice, due to the inherent characteristics of innovative products, consumers have uncertainty in product valuation. At this time, channel preferences not only affect consumers’ purchase methods but also influence manufacturers’ selection of presale entities. Therefore, it is crucial for manufacturers to design a dual-channel presale strategy for innovative products.

This paper explores the presale decision-making for innovative products by different presale entities. By introducing channel preference and product valuation discounts, three game models (the no-presale model, the manufacturer presale model, and the retailer presale model) were constructed to explore their effects on decision variables and supply chain enterprise profits. The conclusions are as follows after comparing the equilibrium results:

1. Compared to no-presale, presales can help manufacturers obtain more profits, and for retailers, the choice of the presale model has a significant impact on their profits. The profit is highest under the retailer presale model, while the profit is lowest under the manufacturer presales model.
2. When the difference in channel preference is small, consumers’ acceptance of the online channel increases, and manufacturers choose the direct presale model to obtain the maximum profit. When the difference in channel preferences is large, the selection of different presale entities has a great effect on product sales and supply chain enterprise profits. Under these conditions, manufacturers should choose retailers to carry out presales. Retailers obtain the most profits when they conduct the presale, and manufacturer presale is detrimental to retailers. Therefore, retailers should actively promote their presale model to obtain more profits.
3. Regardless of whether the valuation of the product is discounted, the profit of the manufacturer from presales is greater than that from the spot sale. When the product valuation discount is high, consumers often tend to buy products during spot sales. At this time, manufacturers can increase the spot sale and wholesale prices of products, while the presale prices remain unchanged to induce consumers to purchase products through presale methods. Therefore, the retailer presale model is more advantageous to manufacturers. When the product valuation discount is low, manufacturers should lower the presale price to improve the presale utility of consumers and encourage consumers to actively participate in presale. In this way, manufacturers’ presale revenue is the highest.
4. When channel preference and the product valuation discount increase simultaneously, there is little difference in the effect of the manufacturer presale model and the retailer presale model on the overall profit of the supply chain. Regardless of which presale model is adopted, it is beneficial for enterprise profit. When both channel preference and the product valuation discount decrease, channel preference decreases and the product valuation discount increases, manufacturers should choose the retailer presale model. When channel preference increases and the product valuation discount decreases, manufacturers should choose the manufacturer presale model.

### 6.2 Managerial insights

The conclusions of this study also provide some managerial insights:

First, information sharing between manufacturers and consumers plays a crucial role in boosting product valuations. By sharing information, the matching degree between the supply and demand of products can be improved, and the shortage rate of products can be reduced, thus alleviating valuation uncertainty caused by out-of-stock.
Second, to improve channel preferences, for online channels, manufacturers should cooperate with retailers to implement a combination of offline experience and online consumption and provide high-quality presale and after-sales services. In addition, manufacturers should also actively respond to consumer concerns about product quality and innovation by providing timely and transparent communication through multiple channels. For offline channels, retailers should provide high-quality customer service and product experience to build consumer trust in the brand.
Third, in view of the competition between channels, manufacturers can use data analysis to better understand consumer behavior and preferences, to provide more accurate products and services in each channel. Adopting differentiation strategies, such as offering slightly different product versions or services in different channels, can diminish direct competition. Manufacturers need to balance competition between online direct sales and offline retail channels to ensure that the profit of the entire supply chain is maximized.

Finally, for manufacturers and retailers in the dual-channel supply chain, before carrying out presale marketing, it is necessary to fully investigate the consumer market, analyze the valuation of consumers for such innovative products, and balance the pros and cons of presale according to the valuation of consumers to determine whether to provide presale services.

This study has certain limitations, such as considering presale decisions for only a single product. This means that we did not consider the competitive relationship between products which may affect the company’s presale strategy. However, in real economic activities, product competition is ubiquitous and can come from both internal and external sources. Therefore, further study can continue from the perspective of product competition. In addition, this study did not consider the situation in which retailers and manufacturers simultaneously engage in presales. For example, if retailers and manufacturers conduct presales simultaneously, they may improve overall sales performance by coordinating their presale strategies at the same time. Hence, future research will need to further expand the model and conclusions to more comprehensively analyze the promotional effect of preselling decisions on corporate innovation and profit increase.

## Supporting information

S1 Appendix (DOC)
