# Supplementary material for: Selection of presale models for innovative products considering channel preferences and valuation differences
Source: PLoS One. 2024 Mar 28;19(3):e0299945. doi: 10.1371/journal.pone.0299945 (PMC10977684; doi:10.1371/journal.pone.0299945)
Supplement: S1 Appendix — (DOC) [file pone.0299945.s001.doc]

**Appendix**

**Proof of Lemma 1.**Since $\frac{\partial^{2}\pi_{r}^{N}\left(p_{s2}^{N}\right)}{\partial\left(p_{s2}^{N}\right)^{2}}=-\frac{2}{1-\theta}<0$, we can see that $\pi_{r}^{N}\left(p_{s2}^{N}\right)$ has a local maximum value. Taking the first-order condition $\frac{\partial\pi_{r}^{N}\left(p_{s2}^{N}\right)}{\partial p_{s2}^{N}}=0$, we can find$p_{s2}^{N}=$ $\frac{1-\theta+p_{s1}^{N}+w_{s2}^{N}}{2}$.Put $p_{s2}^{N}$ into $\pi_{m}^{N}\left(p_{s1}^{N},w_{s2}^{N}\right)$. According to the objective function of the manufacturer, the Hessian matrix is obtained as:$H\left(\pi_{m}^{N}\left(p_{s1}^{N},w_{s2}^{N}\right)\right)=$ $\left|\begin{matrix} -\frac{2-\theta}{\left(1-\theta\right)\theta} & \frac{1}{1-\theta} \\ \frac{1}{1-\theta} & -\frac{1}{1-\theta} \end{matrix}\right|$.The first-order sequential principal minors are$\left|H\left(\pi_{m}^{N}\left(p_{s1}^{N},w_{s2}^{N}\right)\right)\right|=\left|-\frac{2-\theta}{\left(1-\theta\right)\theta}\right|<0$, The second-order sequential principal minor is$\left|H\left(\pi_{m}^{N}\left(p_{s1}^{N},w_{s2}^{N}\right)\right)\right|$ $=\frac{2}{\left(1-\theta\right)\theta}>0$.$H\left(\pi_{m}^{N}\left(p_{s1}^{N},w_{s2}^{N}\right)\right)$ represents a negative definite matrix and $\pi_{m}^{N}\left(p_{s1}^{N},w_{s2}^{N}\right)$is a joint concave function with respect to $p_{s1}^{N}$ and $w_{s2}^{N}$, there exists a unique optimal solution, which can be obtained from the first-order conditions $\frac{\partial\pi_{m}^{N}\left(p_{s1}^{N},w_{s2}^{N}\right)}{\partial p_{s1}^{N}}=0$ and $\frac{\partial\pi_{m}^{N}\left(p_{s1}^{N},w_{s2}^{N}\right)}{\partial w_{s2}^{N}}=0$. Thus, we can obtain the offline wholesale price $w_{s2}^{N*}=\frac{\delta}{2}$and the online retail price$p_{s1}^{N*}=\frac{\theta\delta}{2}$.Put $p_{s1}^{N*}$ and $w_{s2}^{N*}$ into$p_{s2}^{N}$, and we can calculate the optimal offline retail price$p_{s2}^{N*}=\frac{\left(3-\theta\right)\delta}{4}$.

Put the offline wholesale price, the online retail price and the offline retail price into $\pi_{m}^{N}\left(p_{s1}^{N},w_{s2}^{N}\right)$and $\pi_{r}^{N}\left(p_{s2}^{N}\right)$, thus we can obtain the maximum profit for manufacturer and retailer. The proof is completed.

**Proof of Lemma 2.**According to the objective function of the retailer, the Hessian matrix is obtained as$H=\left|\begin{matrix} -\frac{2}{1-\delta} & \frac{2}{1-\delta} \\ \frac{2}{1-\delta} & \frac{2\left(\theta\delta-1\right)}{\left(1-\delta\right)\left(1-\theta\right)\delta} \end{matrix}\right|$.The first-order sequential principal minors are$\left|H\right|=\left|-\frac{2}{1-\delta}\right|<0$, The second-order sequential principal minor is $\left|H\right|=\frac{4}{\left(1-\delta\right)\left(1-\theta\right)\delta}>0$.Thus, $\pi_{r}^{R}\left(p_{a2}^{R},p_{s2}^{R}\right)$is a joint concave function with respect to $p_{a2}^{R}$ and $p_{s2}^{R}$, there exists a unique optimal solution, which can be obtained from the first-order conditions $\frac{\partial\pi_{r}^{R}\left(p_{a2}^{R},p_{s2}^{R}\right)}{\partial p_{a2}^{R}}=0$ and $\frac{\partial\pi_{r}^{R}\left(p_{a2}^{R},p_{s2}^{R}\right)}{\partial p_{s2}^{R}}=0$.Thus, we can obtain the offline presale price and the offline retail price. Put the offline presale price and the offline retail price into the objective function. Similarly, the optimal solution of the manufacturer’s objective function can be obtained.

Put the offline presale price, the wholesale price and the retail price into the objective function, thus we can obtain the maximum profit for manufacturer and retailer. The proof is completed.

**Proof of Proposition 1**

(1)According to Lemma 2,we have $p_{s2}^{R*}-p_{s1}^{R*}=\frac{3\delta\left(1-\theta\right)}{4}>0$ and $p_{a2}^{R*}-p_{s2}^{R*}=\frac{c+3\left(1-\delta\right)}{4}>0$, obviously, $p_{a2}^{R*}>p_{s2}^{R*}>p_{s1}^{R*}$.

(2) We can find $q_{a2}^{R*}+q_{s2}^{R*}=q_{s1}^{R*}=\frac{1}{4}$, therefore , it is easy to get $q_{a2}^{R*}<q_{s1}^{R*}$ and $q_{s2}^{R*}<q_{s1}^{R*}$.

According to Lemma 2,we have $q_{a2}^{R*}-q_{s2}^{R*}=\frac{1-\delta-2c}{4\left(1-\delta\right)}$,when$\frac{1-\delta}{2}<c\leq1-\delta$,we can obtain$q_{a2}^{R*}<q_{s2}^{R*}$; otherwise, $q_{a2}^{R*}>q_{s2}^{R*}$.The proof is completed.

**Proof of Lemma 3.** Since $\frac{\partial^{2}\pi_{r}^{M}\left(p_{s2}^{M}\right)}{\partial\left(p_{s2}^{M}\right)^{2}}=-\frac{2}{\left(1-\theta\right)\delta}<0$, we can see that $\pi_{r}^{M}\left(p_{s2}^{M}\right)$ has a local maximum value. Taking the first-order condition $\frac{\partial\pi_{r}^{M}\left(p_{s2}^{M}\right)}{\partial p_{s2}^{M}}=0$, we can find$p_{s2}^{M}=$ $\frac{\left(\left(w_{s2}^{M}+p_{a1}^{M}\right)\theta-p_{a1}^{M}+p_{s1}^{M}\right)\delta-\left(p_{s1}^{M}+w_{s2}^{M}\right)\theta}{2\theta\left(\delta-1\right)}$. Put $p_{s2}^{M}$into $\pi_{m}^{M*}$. According to the objective function of the manufacturer, the Hessian matrix is obtained as:$H=\left|\begin{matrix} -\frac{2}{\left(1-\delta\right)\theta} & \frac{3}{2\left(1-\delta\right)\theta} & \frac{1}{2\left(1-\delta\right)\theta} \\ \frac{3}{2\left(1-\delta\right)\theta} & \frac{\delta+\theta-2}{\left(1-\delta\right)\left(1-\theta\right)\theta\delta} & \frac{2\theta-\left(1+\theta\right)\delta}{2\left(1-\delta\right)\left(1-\theta\right)\theta\delta} \\ \frac{1}{2\left(1-\delta\right)\theta} & \frac{2\theta-\left(1+\theta\right)\delta}{2\left(1-\delta\right)\left(1-\theta\right)\theta\delta} & \frac{1}{\left(\theta-1\right)\delta} \end{matrix}\right|$.The first-order sequential principal minors are$\left|H\right|=\left|-\frac{2}{\left(1-\delta\right)\theta}\right|<0$.The second-order sequential principal minor is $\left|H\right|=\frac{9\theta\delta-17\delta-8\theta+16}{4\theta^{2}\left(1-\delta\right)^{2}\left(1-\theta\right)\delta}>0$. The third-order sequential principal minor is $\left|H\right|=\frac{7\theta\delta+\delta-8\theta}{2\theta^{3}\left(1-\delta\right)^{2}\left(1-\theta\right)\delta^{2}}<0$.There exists a unique optimal solution, we can obtain$w_{s2}^{M*}=\frac{\left[\left(4-3\delta\right)\theta^{2}+\left(4-4c-5\delta\right)\theta+4c\right]\delta}{2\left[8\theta-\left(1+7\theta\right)\delta\right]}$,$p_{s1}^{M*}=\frac{\theta\delta}{2}$,$p_{a1}^{M*}=\frac{\left[8\left(c+\theta\right)\left(1-\delta\right)-\left(1-\theta\right)\delta^{2}\right]\theta}{2\left[8\theta-\left(1+7\theta\right)\delta\right]}$.Put $\left(w_{s2}^{M*},p_{s1}^{M*},p_{a1}^{M*}\right)$into $p_{s2}^{M}$. we can obtain $p_{s2}^{M*}=\frac{\left[\left(2-\delta\right)\theta^{2}+\left(6-6c-7\delta\right)\theta+6c\right]\delta}{2\left[8\theta-\left(1+7\theta\right)\delta\right]}$.Put the offline presale price, the wholesale price and the retail price into the objective function, thus we can obtain the maximum profit for manufacturer and retailer. The proof is completed.

**Proof of Proposition 2**

(1)We have $p_{s2}^{M*}-p_{a1}^{M*}=\frac{\left[\left(1-\delta\right)\theta+c\right]\left[\left(\delta-4\right)\theta+3\delta\right]}{8\theta-\left(1+7\theta\right)\delta}$.According to Lemma 3, we have $\delta<$ $\frac{8\theta}{1+7\theta}$. Because $\left(\delta-4\right)\theta+3\delta>\frac{8\theta}{1+7\theta}\left(\theta+3\right)-4\theta=\frac{20\theta\left(1-\theta\right)}{1+7\theta}>0$, we can obtain $p_{s2}^{M*}>p_{a1}^{M*}$. In addition,$p_{s2}^{M*}-p_{s1}^{M*}=\frac{3\delta\left(\theta+c-\theta\delta\right)\left(1-\theta\right)}{8\theta-\left(1+7\theta\right)\delta}>0$ and $p_{a1}^{M*}-p_{s1}^{M*}=\frac{4\theta\left(\theta+c-\theta\delta\right)\left(1-\delta\right)}{8\theta-\left(1+7\theta\right)\delta}>0$, thus, $p_{s2}^{M*}>p_{s1}^{M*}$and $p_{a1}^{M*}>p_{s1}^{M*}$.

(2)According to Lemma 3, we have $q_{s1}^{M*}-q_{a1}^{M*}=\frac{\left(7\theta+3\right)\delta+14c-10\theta}{2\left[8\theta-\left(1+7\theta\right)\delta\right]}$.When $q_{s1}^{M*}=q_{a1}^{M*}$, we can obtain$c_{2}=\frac{10\theta-\left(7\theta+3\right)\delta}{14}$.Similarly, it is easy to get : when $q_{s2}^{M*}=q_{a1}^{M*}$, $c3=\frac{3\theta-\left(2\theta+1\right)\delta}{5}$.

When $q_{s1}^{M*}-q_{s2}^{M*}=\frac{1}{2}q_{a1}^{M*}=\frac{\left(4-3\delta\right)\theta-4c-\delta}{2\left[8\theta-\left(1+7\theta\right)\delta\right]}=0$,$c_{1}=\frac{4\theta-\left(3\theta+1\right)\delta}{4}$.

While $c_{1}-c_{2}=\frac{8\theta-\left(1+7\theta\right)\delta}{28}>0$,$c_{1}-c_{3}=\frac{8\theta-\left(1+7\theta\right)\delta}{20}>0$,$c_{3}-c_{2}=-\frac{8\theta-\left(1+7\theta\right)\delta}{70}<0$, thus$c_{3}<c_{2}<c_{1}$. In addition, we have $c<\frac{4\theta-\left(3\theta+1\right)\delta}{4}$, obviously $q_{s1}^{M*}-q_{s2}^{M*}<0$. The proof is completed.

**Proof of Proposition 3.**

(1) From Lemma 2, it is easy to get $\frac{\partial p_{a2}^{R*}}{\partial\theta}=-\frac{\delta}{4}<0$,$\frac{\partial p_{s2}^{R*}}{\partial\theta}=-\frac{\delta}{4}<0$,$\frac{\partial p_{s1}^{R*}}{\partial\theta}=\frac{\delta}{2}>0$,$\frac{\partial\pi_{m}^{R*}}{\partial\theta}=\frac{\delta}{8}>0$.

(2)According to Lemma 3,

$\frac{\partial p_{a1}^{M*}}{\partial\theta}=\frac{64\theta^{2}+\left(1-7\theta^{2}-2\theta\right)\delta^{3}+\left(64\theta^{2}+8c+16\theta\right)\delta^{2}-\left(120\theta^{2}+8c+16\theta\right)\delta}{2\left(8\theta-\left(1+7\theta\right)\delta\right)^{2}}$.We have$\delta<\frac{8\theta}{1+7\theta}$, obviously$\frac{\partial p_{a1}^{M*}}{\partial\theta}>\frac{\left(1+\theta\right)\delta\left(\delta-1\right)+8\theta-\left(1+7\theta\right)\delta}{2\left(8\theta-\left(1+7\theta\right)\delta\right)}>0$. Besides, it is easy to prove$\frac{\partial p_{s1}^{M*}}{\partial\theta}=\frac{\delta}{2}>0$,$\frac{\partial q_{s2}^{M*}}{\partial\theta}=\frac{\delta^{2}+\left(7c-1\right)\delta-8c}{\left(8\theta-\left(1+7\theta\right)\delta\right)^{2}}=\frac{\left(7\delta-8\right)c+\delta\left(\delta-1\right)}{\left(8\theta-\left(1+7\theta\right)\delta\right)^{2}}<0$,$\frac{\partial q_{a1}^{M*}}{\partial\theta}=-\frac{4\partial q_{s2}^{R*}}{\partial\theta}>0$,$\frac{\partial q_{s1}^{M*}}{\partial\theta}=\frac{3\partial q_{s2}^{M*}}{\partial\theta}<0$.

We assume $\frac{\partial p_{s2}^{M*}}{\partial\theta}=\frac{\left(7+7\theta^{2}+2\theta\right)\delta^{3}+\left(-22\theta^{2}+48c-4\theta-6\right)\delta^{2}+\left(16\theta^{2}-48c\right)\delta}{2\left(8\theta-\left(1+7\theta\right)\delta\right)^{2}}=0$,and after simplification we can obtain $\overline{\theta}=\frac{\left(2-\delta\right)+4\sqrt{3\left(1-\delta\right)\left(2-\delta\right)\left(8c+\delta-7c\delta-\delta^{2}\right)}}{\left(2-\delta\right)\left(8-7\delta\right)}$.when $\frac{1}{2}<\theta<\overline{\theta}$，$\frac{\partial p_{s2}^{M*}}{\partial\theta}<0$,otherwise, $\frac{\partial p_{s2}^{M*}}{\partial\theta}>0$. The proof is completed.

**Proof of Proposition 4.**

(1)$\frac{\partial\pi_{r}^{R*}}{\partial\theta}=-\frac{\delta}{16}<0$;$\frac{\partial p_{a2}^{R*}}{\partial\delta}=-\frac{\theta}{4}<0$,$\frac{\partial p_{s2}^{R*}}{\partial\delta}=\frac{3-\theta}{4}>0$,$\frac{\partial p_{s1}^{R*}}{\partial\delta}=\frac{\theta}{2}>0$,$\frac{\partial\pi_{m}^{R*}}{\partial\delta}=\frac{\left(1-\delta\right)^{2}\theta+c^{2}}{8\left(1-\delta\right)^{2}}>0$.

From Equation (4), $\frac{\partial\pi_{r}^{R*}}{\partial\delta}=\frac{c^{2}-\left(1-\delta\right)^{2}\theta}{16\left(1-\delta\right)^{2}}$ ,when $1-\frac{c}{\sqrt{\theta}}\leq\delta\leq1-c$, obviously $\frac{\partial\pi_{r}^{R*}}{\partial\delta}>0$, otherwise, $\frac{\partial\pi_{r}^{R*}}{\partial\delta}<0$.

(2)According to Lemma 3,$\frac{\partial p_{a1}^{M*}}{\partial\delta}=\frac{\theta\left(1-\theta\right)\left(\left(7\delta^{2}-16\delta+8\right)\theta+\delta^{2}+8c\right)}{2\left(8\theta-\left(1+7\theta\right)\delta\right)^{2}}$,we have$\left(7\delta^{2}-16\delta+8\right)\theta$ $+\delta^{2}+8c=\left(8\left(1-\delta\right)^{2}-\delta^{2}\right)\theta+\delta^{2}+c>0$, obviously $\frac{\partial p_{a1}^{M*}}{\partial\delta}>0$.Therefore , it is easy to get:

$\frac{\partial p_{s1}^{M*}}{\partial\delta}=\frac{\theta}{2}>0$,$\frac{\partial p_{s2}^{M*}}{\partial\delta}=\frac{\left(7\delta^{2}+16-16\delta\right)\theta^{2}+\left(48+50\delta^{2}-48c-102\delta\right)\theta+7\delta^{2}+48c}{2\left(8\theta-\left(1+7\theta\right)\delta\right)^{2}}>-$ $\frac{\theta\left(\left(\delta+4\right)\theta+7\delta-12\right)}{2\left(8\theta-\left(1+7\theta\right)\delta\right)}>0$,$\frac{\partial q_{a1}^{M*}}{\partial\delta}=\frac{4\theta^{2}-4\left(1+7c\right)\theta-c}{\left(8\theta-\left(1+7\theta\right)\delta\right)^{2}}<0$,$\frac{\partial q_{s2}^{M*}}{\partial\delta}=\frac{\left(1+7\theta\right)c+\theta\left(1-\theta\right)}{\left(8\theta-\left(1+7\theta\right)\delta\right)^{2}}>0$,$\frac{\partial q_{s1}^{M*}}{\partial\delta}=\frac{1}{3}\frac{\partial q_{s2}^{M*}}{\partial\delta}>0$. The proof is completed.

**Proof of Proposition 5.**$q_{s1}^{N*}+q_{s2}^{N*}=\frac{1}{2}$,$q_{a1}^{M*}+q_{s1}^{M*}+q_{s2}^{M*}=\frac{1}{2}$,$q_{s1}^{R*}+q_{a2}^{R*}+q_{s2}^{R*}=\frac{1}{2}$.The proof is completed.

**Proof of Proposition 6.**

(1)According to Lemma 2 and Lemma 3, we have$p_{s2}^{M*}-p_{s2}^{R*}=-\frac{3\delta\left[\left(4-3\delta\right)\theta-4c-\delta\right]\left(1-\theta\right)}{4\left[8\theta-\left(1+7\theta\right)\delta\right]}$ $<0$, thus ,$p_{s2}^{M*}<p_{s2}^{R*}$.

Similarly,$p_{a1}^{M*}-p_{a2}^{R*}=\frac{-\left(5\delta^{2}+8\delta-16\right)\theta^{2}-\left(3\delta^{2}-21\delta+24\right)\theta+3\delta-9c\delta\theta+8c\theta+c\delta}{4\left[8\theta-\left(1+7\theta\right)\delta\right]}$, since$8\theta+\delta-9\delta\theta>0$ and $c<\frac{4\theta-\left(3\theta+1\right)\delta}{4}$, we can find $p_{a1}^{M*}-p_{a2}^{R*}<-\frac{\left(1-\theta\right)\left(12-\delta\right)}{16}<0$.

(2)Similarly, $q_{s1}^{M*}-q_{s1}^{R*}=\frac{9\theta\delta+3\delta+12c-12\theta}{4\left[8\theta-\left(1+7\theta\right)\delta\right]}$,we have $c<\frac{4\theta-\left(3\theta+1\right)\delta}{4}$, therefore,$q_{s1}^{M*}-q_{s1}^{R*}>0$.

According to Lemma 1 and Lemma 2, we have $q_{s2}^{R*}-q_{s2}^{N*}=\frac{c+\delta-1}{4\left(1-\delta\right)}$, since $1-\delta-c>0$, thus $q_{s2}^{R*}<q_{s2}^{N*}$.Similarly,$q_{s2}^{R*}-q_{s2}^{M*}=\frac{\left[\left(8-7\delta\right)\theta-\left(4-3\delta\right)\right]c-4\delta^{2}\theta+8\theta\delta-4\theta}{4\left(1-\delta\right)\left[8\theta-\left(1+7\theta\right)\delta\right]}$，we have $\theta>\frac{1}{2}$and $c<\frac{4\theta-\left(3\theta+1\right)\delta}{4}$, obviously,$\left(8-7\delta\right)\theta-\left(4-3\delta\right)>0$,Therefore,$q_{s2}^{R*}-q_{s2}^{M*}<-\frac{\left(4-3\delta\right)\left(1-\theta\right)}{16\left(1-\delta\right)}$ $<0$and $q_{s2}^{M*}-q_{s2}^{N*}=$ $-\frac{\left(4-3\delta\right)\theta-4c-\delta}{4\left[8\theta-\left(1+7\theta\right)\delta\right]}<0$,we can obtain $q_{s2}^{N*}>q_{s2}^{M*}>q_{s2}^{R*}$.

We have$q_{a1}^{M*}-q_{a2}^{R*}=\frac{\left(16-15\delta-8\theta+7\theta\delta\right)c-\left(5\theta+3\right)\delta^{2}+13\theta\delta+3\delta-8\theta}{4\left(1-\delta\right)\left[8\theta-\left(1+7\theta\right)\delta\right]}$, since $16-15\delta-8\theta$ $+7\theta\delta>0$, so $q_{a1}^{M*}-q_{a2}^{R*}<-\frac{\left(4-3\delta\right)\left(1-\theta\right)}{16\left(1-\delta\right)}<0$.The proof is completed.
